# RNA Editing in Chloroplasts of *Spirodela polyrhiza*, an Aquatic Monocotelydonous Species

**DOI:** 10.1371/journal.pone.0140285

**Published:** 2015-10-30

**Authors:** Wenqin Wang, Wei Zhang, Yongrui Wu, Pal Maliga, Joachim Messing

**Affiliations:** Waksman Institute of Microbiology, Rutgers, The State University of New Jersey, Piscataway, New Jersey, United States of America; Agriculture and Agri-Food Canada, CANADA

## Abstract

RNA editing is the post-transcriptional conversion from C to U before translation, providing a unique feature in the regulation of gene expression. Here, we used a robust and efficient method based on RNA-seq from non-ribosomal total RNA to simultaneously measure chloroplast-gene expression and RNA editing efficiency in the Greater Duckweed, *Spirodela polyrhiza*, a species that provides a new reference for the phylogenetic studies of monocotyledonous plants. We identified 66 editing sites at the genome-wide level, with an average editing efficiency of 76%. We found that the expression levels of chloroplast genes were relatively constant, but 11 RNA editing sites show significant changes in editing efficiency, when fronds turn into turions. Thus, RNA editing efficiency contributes more to the yield of translatable transcripts than steady state mRNA levels. Comparison of RNA editing sites in coconut, Spirodela, maize, and rice suggests that RNA editing originated from a common ancestor.

## Introduction

RNA editing in angiosperms mainly defines the process that alters a cytosine (C) to uracil (U) in specific positions of RNA so that the sequence in the mature RNA differs from that of genomic DNA. RNA editing is a mechanism that corrects missense mutations of genes at the RNA level. It thereby restores conserved amino acid residues to maintain essential functions of encoded proteins [1]. For example, *psbF* mRNA is edited in spinach plastids by a C to U conversion, changing a serine to a conserved phenylalanine codon. In tobacco, a phenylalanine codon is present at the DNA level without any editing. When the spinach *psbF* was introduced into tobacco plastids, the lack of RNA editing led to a defective phenotype, indicating that RNA editing is site-specific [2]. Introduction of the tobacco chloroplast genome into *Atropa belladonna*, demonstrates that the belladonna nuclear genome is unable to edit the tobacco plastid ATPase α-subunit transcript, resulting in an albino phenotype of the cytoplasmic hybrid plant [3].

RNA editing is absent in algae and highly abundant (300–500 sites) in hornworts and ferns, but unusually high in lycophytes, where 3,415 RNA-editing events were found [4]. In Cycas, 85 editing sites have been identified in 25 transcripts [5]. Editing sites decrease to 35 ~ 41 in more recently emerging monocotyledonous (monocots) and dicotyledonous (dicots) species [1,5,6]. Given the degree of conservation of editing sites among three dicots, one monocot, one gymnosperm, one fern and one hornwort, the evolution of chloroplast RNA editing is hypothesized to be of monophyletic origin [7]. Other investigations also suggest that RNA editing shares a common ancestor in the same species family. For example, maize, rice and sugarcane within the Poaceae family share 23 out of a total of 25 editing sites [8].

However, to validate such a hypothesis would require RNA editing studies for species spread throughout the phylogenetic tree. The genomes of most monocot species that have been studied are from the clade of the Commelinids and therefore do not provide a genome of a more distantly related species of monocot plants.

Recently, we studied the nuclear and chloroplast genomes of the aquatic plant Spirodela (*Spirodela polyrhiza*), belonging to the subfamily of Lemnoideae in the order of Alismatales [9,10]. Spirodela, as a basal monocot, has a very unique life cycle [11]. In its growth phase, leaf-like structures (fronds) reproduce by clonal budding under optimal conditions. In their dormant phase it forms turions, which produce starch and secondary metabolites like seeds, when there is shortage of nutrition in the fall or the temperature drops in the winter [10,12]. Therefore, the genome of Spirodela not only provides a new evolutionary reference point for monocots, but also a species that is exposed to very different environmental conditions than terrestrial plants.

In this context, the question arises to which degree RNA editing evolved in Lemnoideae as a distinct clade of basal monocots. Critical to this question is the quantification of chloroplast RNA editing efficiency in different tissue types. There could be three main mechanisms that regulate the levels of edited mRNAs. One is the control of gene expression at the transcriptional level and second the modulation of RNA editing efficiency at the post-transcriptional level. A third one is the mechanism of RNA turnover. An increased rate of RNA degradation, leading to a reduced half-life for the RNA, reduces the proportion of edited RNA. On the other hand, more efficient RNA editing elevates edited transcript levels, which in turn may produce more functional proteins. There are many other factors involved in the yield of chloroplast proteins, such as protein translation, protein stability and protein import efficiencies into organelles. Here, we only focus on understanding the relative importance of editing efficiency and transcript abundance on the yield of edited transcripts, thus, we have monitored the extent of editing at each identified site.

## Materials and Methods

### Read mapping and SNP calling

Due to the high coverage of chloroplast transcript reads from total RNA sequencing [13] we could use very stringent quality control parameters regarding abundant organelle sequences; i.e. reads with an average score > = 20 and length > = 70bp were applied. The raw data can be downloaded from the BioProject PRJNA205940 with accession numbers of SAMN02355992 ~SAMN02355999.

The RNA-seq reads were mapped strand specifically with BWA [14] to its chloroplast genome (GenBank Accession #JN160603), which was sequenced and assembled from total DNA [9]. After mapping, we determined the relative amounts of transcripts for protein coding genes with their FPKM values (fragments per kilobase of exon per million mapped reads). We further defined the differentially expressed genes if the fold change was more than 2 and false discovery rate (FDR) was less than 0.05. SNPs were called by SAMtools to uncover potential changes of C to U when at a given position with coverage limit set to 10 reads. The coverage setting is arbitrary, but it is sufficient to identify all potential SNPs, while excluding sequencing errors at the same time. We took advantage of the four biological replicates and only kept the SNPs that were present in at least two replicates. The mapped reads were then visualized in the Integrative Genomics Viewer [15]. RNA editing efficiency was counted by edited reads divided by total mapped reads. The Chi-squared test was used to determine edited sites with significant changes.

### RNA editing validation

The EST sequences from total RNA including nuclear and organellar sequences were downloaded from the NCBI Sequence Read Archive (SRA), submitted by the DOE Joint Genome Institute (JGI) from our 454 sequencing of Spirodela ESTs (SRX148325). For unconfirmed sites, we designed specific primers spanning the candidate regions and performed RT-PCR; the resulting products were directly sequenced using the same PCR primers. These sites were confirmed as RNA editing sites only if there were two overlapping peaks at the same location. To further validate editing efficiency, we cloned the RT-PCR products into pGEM-T easy and selected 96 clones for sequencing to validate the edited sites by comparing them with the genomic DNA sequence of Spirodela.

### Database for chloroplast genome

All chloroplast genome sequences used in this study were downloaded from Genbank: Spirodela JN160603; coconut KF285453; rice NC_001320; maize NC_001666; tobacco NC_001879; Arabidopsis NC_000932. The editing sites for coconut [16], rice [17], maize [18], tobacco [19] and Arabidopsis [20] were collected from respective publications. All sequence alignments were performed with clustalW and a phylogenetic tree was constructed with MEGA6 [21]. To simplify our presentation, the common names were used in main texts, tables and figures. Their scientific names are: Spirodela—*Spirodela polyrhiza*; coconut—*Cocos nucifera*; rice–*Oryza sativa*; maize—*Zea mays*; tobacco—*Nicotiana tabacum*; Arabidopsis—*Arabidopsis thaliana*; tomato–*Solanum lycopersicum*.

## Results

### Mapping statistics and chloroplast gene expression

**Fig 1:**
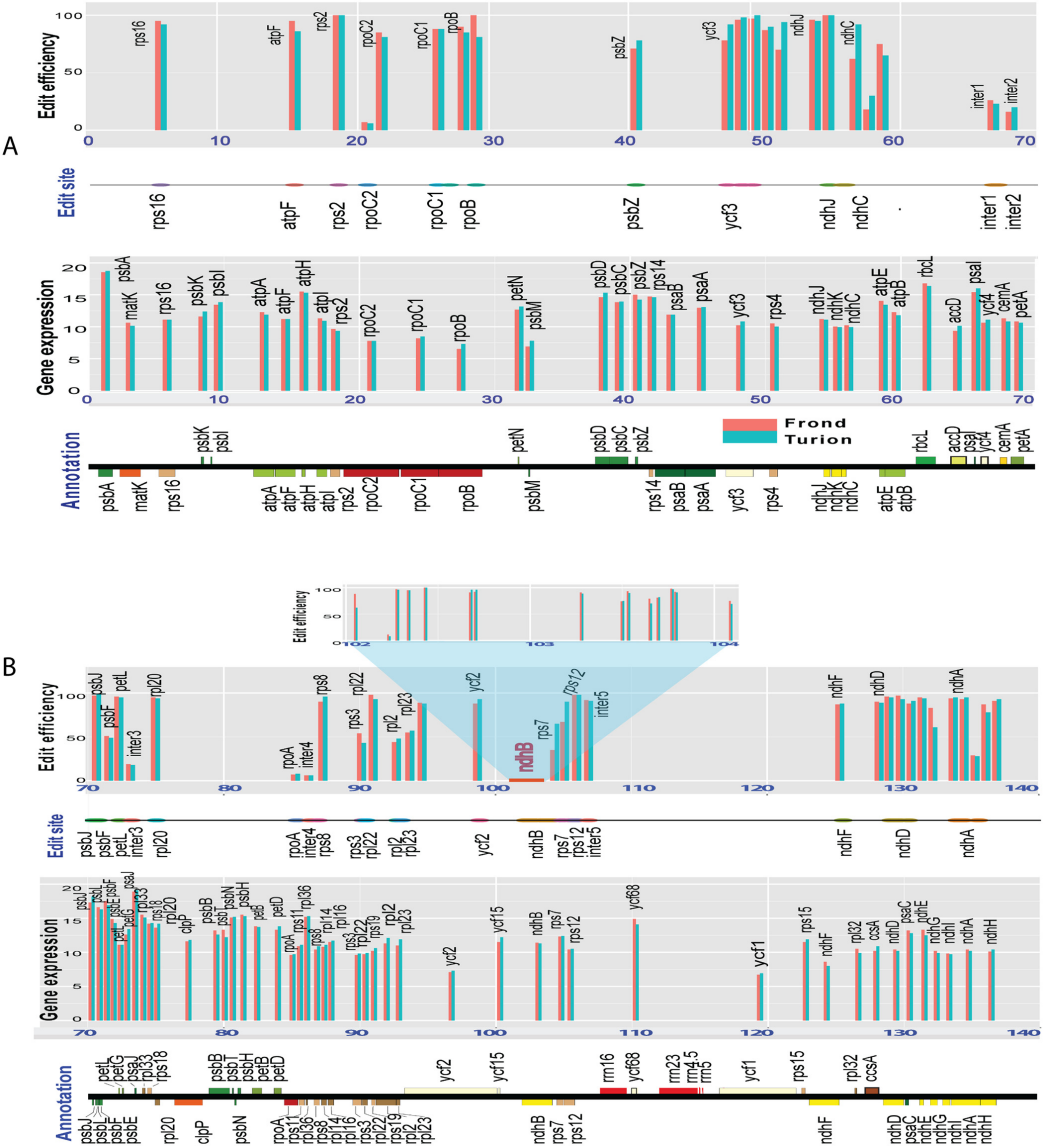
Overview of chloroplast gene expression, RNA editing sites and corresponding editing efficiencies. Annotation from Genbank accession JN160603 was viewed with OGDRAW [22]. Only one of the inverted repeats is shown here. The X-axis shows the genome position. Chloroplast gene expression is shown as the value of log2 (FPKM). Gene expression and RNA editing sites were matched to the genomic position, whereas the bars for editing efficiency (e.g *ndhD*) were shifted due to multiple editing sites within very narrow windows. The *ndhB* gene (15 sites) was thus drawn in a separate window. Layers were counted from the bottom up. Layer 1: Annotation; Layer 2: Gene expression; Layer 3: RNA editing sites; Layer 4: RNA editing efficiency; Layer 5 (only in part B): *ndhB* editing efficiency. To clearly oversee genome-wide RNA editing, the chloroplast genome was split into two 70Kbp fragments. A) First 70 Kbp of chloroplast genome; B) Second 70 Kbp of chloroplast genome.

Our previous study showed that more than 26% of the RNA-seq reads from ribosomal depleted total RNAs could be mapped back to the chloroplast genome, equal to ~2,000-fold deep coverage [13]. Here, we used a stringent filter to analyze only reads with a score of 20 and minimal length of 70 bp. A range of 1,315,402 to 4,109,489 reads equal to ~1,000-fold coverage was mapped back to the chloroplast genome, which collectively represented more than 20% of the total reads (S1 Table). Read density varied widely between different genomic regions, which reflected the differential accumulation of chloroplast RNA. For example, *psbA*, *rbcL* and *psaJ* were highly expressed, whereas *rpoC2*, *rpoC1* and *rpoB* were expressed at low levels (Fig 1). However, for the individual gene, the FPKM value did not change significantly, when turion formation was induced, suggesting that chloroplast genes were expressed at a constant level as fronds developed into turions (S2 Table).

### Detection of chloroplast RNA editing sites

The high read density at most sites allowed us to assess a robust qualification and quantification of editing events. A total of 66 RNA editing sites of C-to-U conversion from 27 genes were found, when they were transcribed (Fig 1 and Table 1). All sites were validated either with 454 or traditional capillary electrophoresis (CE) platforms, where two overlapping peaks (C and T) were seen at RNA editing sites. The most heavily edited genes were *ndhB* (15 sites), *ndhD* (6 sites) and *ndhA* (5 sites). As expected, the RNA editing in the first and second position of codons changes the identity of amino acid, whereas it was silent for the third codon. Of the 58 editing sites in protein coding regions, 6 sites (10.3%) were in first, 49 sites (84.5%) in second, and three sites (5.2%) in third codons. The conversions from ACG to AUG in *rpl2* and *ndhD* genes were found to create an initiation codon in Spirodela. Due to the depth of sequencing, we also detected eight silent RNA editing sites (12.1% from total) in non-coding sequences that have been rarely reported for chloroplast genome (S3 and S4 Tables) with the exception of the report in Arabidopsis [23]. Two sites were located in the intron of *ycf3* and *ndhB*, one in the 5’ UTR of *rps7*, and five in intergenic regions. RNA editing sites from UTR, introns, or intergenic regions provide us perhaps with a new evolutionary cause for RNA editing, as we do not know whether these editing events contribute to essential functions of plastids.

**Table 1:** List of RNA editing sites in the chloroplast of Spirodela.

Gene	Genome Position	Gene Position	Codon	Amino Acid	Edit (%) Fronds	Edit (%) Turions	Cn^a^	Os^b^	Zm^c^	At^d^	Nt^e^	Sl^f^
5'UTR rps7-1	105389	-	-	-	67%	90%	?	?	?	T	T	T
atpF	15203	92	cCa	P>L	95%	86%	+	T	T	+	+	?
intergenic-1	66923	-	-	-	26%	23%	NA	NA	NA	NA	NA	NA
intergenic-2	67274	-	-	-	16%	20%	NA	NA	NA	NA	NA	NA
intergenic-3	73142	-	-	-	19%	18%	NA	NA	NA	NA	NA	NA
intergenic-4	86259		-	-	6%	6%	?	?	?	?	?	?
intergenic-5	106653	-	-	-	92%	91%	?	T	T	T	T	T
ndhA-1	135335	476	uCa	S>L	91%	93%	+	+	+	T	T	T
ndhA-2	135281	530	cCa	P>L	87%	78%	T	T	T	T	T	T
ndhA-3	134193	566	uCa	S>L	29%	28%	+	?	+	T	T	T
ndhA-4	133807	952	Cct	P>S	93%	95%	+	T	T	T	?	?
ndhA-5	133695	1064	uCc	S>F	94%	95%	+	+	+	T	+	?
ndhB-1	104089	149	uCa	S>L	75%	69%	+	T	T	+	+	+
ndhB-2	103792	446	uCa	S>L	92%	91%	T	T	T	T	T	T
ndhB-3	103771	467	cCa	P>L	98%	97%	+	+	+	+	+	+
ndhB-4	103696	542	aCg	T>M	81%	82%	+	T	T	T	T	T
ndhB-5	103652	586	Cau	H>Y	79%	70%	+	+	+	+	+	+
ndhB-6	103534	704	uCc	S>F	93%	90%	+	+	T	T	T	T
ndhB-7	103501	737	cCa	P>L	74%	75%	+	+	+	T	+	+
ndhB-8 intron	103280	-	-	-	91%	89%	?	?	?	?	?	?
ndhB-9	102704	830	uCa	S>L	92%	96%	+	+	+	+	+	+
ndhB-10	102698	836	uCa	S>L	91%	96%	+	+	T	+	+	+
ndhB-11	102432	1102	Cgc	R>C	100%	100%	T	T	T	T	T	T
ndhB-12	102341	1193	uCa	S>L	95%	95%	+	T	T	T	T	T
ndhB-13	102279	1255	Cau	H>Y	97%	96%	+	T	T	+	T	T
ndhB-14	102233	1301	uCa	S>L	11%	8%	?	?	?	?	T	T
ndhB-15	102053	1481	cCa	P>L	88%	62%	+	+	+	+	+	+
ndhC-1	56026	13	Cac	H>Y	75%	65%	?	?	?	T	T	T
ndhC-2	55728	311	cCa	P>L	18%	30%	?	T	T	T	T	T
ndhC-3	55716	323	uCa	S>L	62%	92%	?	T	T	T	T	T
ndhD-1	130160	2	aCg	T>M	83%	61%	+	T	T	+	+	+
ndhD-2	129488	674	uCa	S>L	95%	94%	+	T	T	+	+	+
ndhD-3	129284	878	uCa	S>L	88%	91%	T	+	+	+	T	+
ndhD-4	129215	947	aCa	T>I	97%	93%	+	T	T	T	T	T
ndhD-5	128969	1193	uCa	S>L	96%	95%	+	T	T	T	T	T
ndhD-6	128852	1310	uCa	S>L	90%	89%	+	T	T	T	+	+
ndhF	125342	62	uCa	S>L	87%	88%	+	+	+	T	T	T
ndhJ-1	54835	10	Cau	H>Y	100%	100%	?	T	T	NA	?	?
ndhJ-2	54717	128	uCa	S>L	96%	95%	?	T	T	T	T	T
petL	72275	44	uCa	S>L	96%	95%	T	T	T	T	T	T
psbF	70715	77	uCu	S>F	51%	49%	T	T	T	?	T	T
psbJ	70337	71	uCa	S>L	97%	99%	T	T	T	T	T	T
psbZ	40491	50	uCa	S>L	71%	78%	?	?	?	+	T	T
rpl2	92708	2	aCg	T>M	44%	48%	?	+	+	T	T	T
rpl20	74981	308	uCg	S>L	95%	94%	+	T	+	T	+	?
rpl22	90376	233	uCa	S>L	98%	93%	?	T	T	T	T	T
rpl23-1	92939	71	uCu	S>F	89%	88%	+	T	T	T	?	?
rpl23-2	92921	89	uCa	S>L	55%	57%	+	T	T	?	?	?
rpoA	85276	200	uCu	S>F	7%	8%	+	T	T	?	?	?
rpoB-1	28650	473	uCg	S>L	100%	81%	+	?	+	T	+	+
rpoB-2	26691	2432	uCa	S>L	90%	85%	+	T	T	+	T	+
rpoC1	25833	62	cCa	P>L	88%	88%	+	T	T	T	+	?
rpoC2-1	20639	2318	uCa	S>L	85%	81%	?	T	+	T	T	T
rpoC2-2	20579	2378	cCa	P>L	7%	6%	?	?	?	?	?	?
rps12	105777	221	uCa	S>L	98%	98%	T	T	T	T	T	+
rps16	5284	143	uCa	S>L	95%	92%	T	T	T	T	T	T
rps2	18456	134	aCa	T>I	100%	100%	+	?	+	T	+	?
rps3	90143	30	uuC	I>I	54%	43%	?	?	?	?	?	?
rps7-2	104732	300	gcC	A>A	35%	65%	?	?	?	?	?	?
rps8	86875	182	uCa	S>L	90%	96%	+	+	+	T	T	T
ycf2	98676	5354	uCa	S>L	88%	93%	T	NA	NA	T	T	T
ycf3-1	49098	63	atC	I>I	70%	94%	T	T	T	?	?	?
ycf3-2 intron	48447	-	-	-	87%	90%	?	?	?	T	T	T
ycf3-3	48230	185	aCg	T>M	97%	100%	+	?	+	T	T	T
ycf3-4	48224	191	cCa	P>L	96%	98%	+	T	T	T	T	T
ycf3-5	47241	407	uCc	S>F	78%	92%	+	T	T	T	T	T

“Genome Position” means the location of RNA editing in the genome. “Gene Position” means the location of RNA editing in the gene. "Edit (%)” gives the percentage of RNA editing using the edited reads divided by total mapped reads. The comparison of Spirodela with other model plants of coconut, rice, maize, Arabidopsis, tobacco and tomato was also listed. “T” means pre-edited T at the DNA level (no editing at RNA level); “+” means experimentally determined editing sites; the “?” means the potential editing site due to the existence of “C”; “-” means no editing in spite of C present in the genome.

^a^Cn—*Cocos nucifera*;

^b^Os–*Oryza sativa*;

^c^Zm—*Zea mays*;

^d^At—*Arabidopsis thaliana*;

^e^Nt—*Nicotiana tabacum*;

^f^Sl–*Solanum lycopersicum*.

### RNA editing evolution in monocots

Compared with other angiosperms, the number of RNA editing events in Spirodela (66) and coconut (75) were about twice the editing sites of rice (35) and maize (26) [1,24].

**Fig 2:**
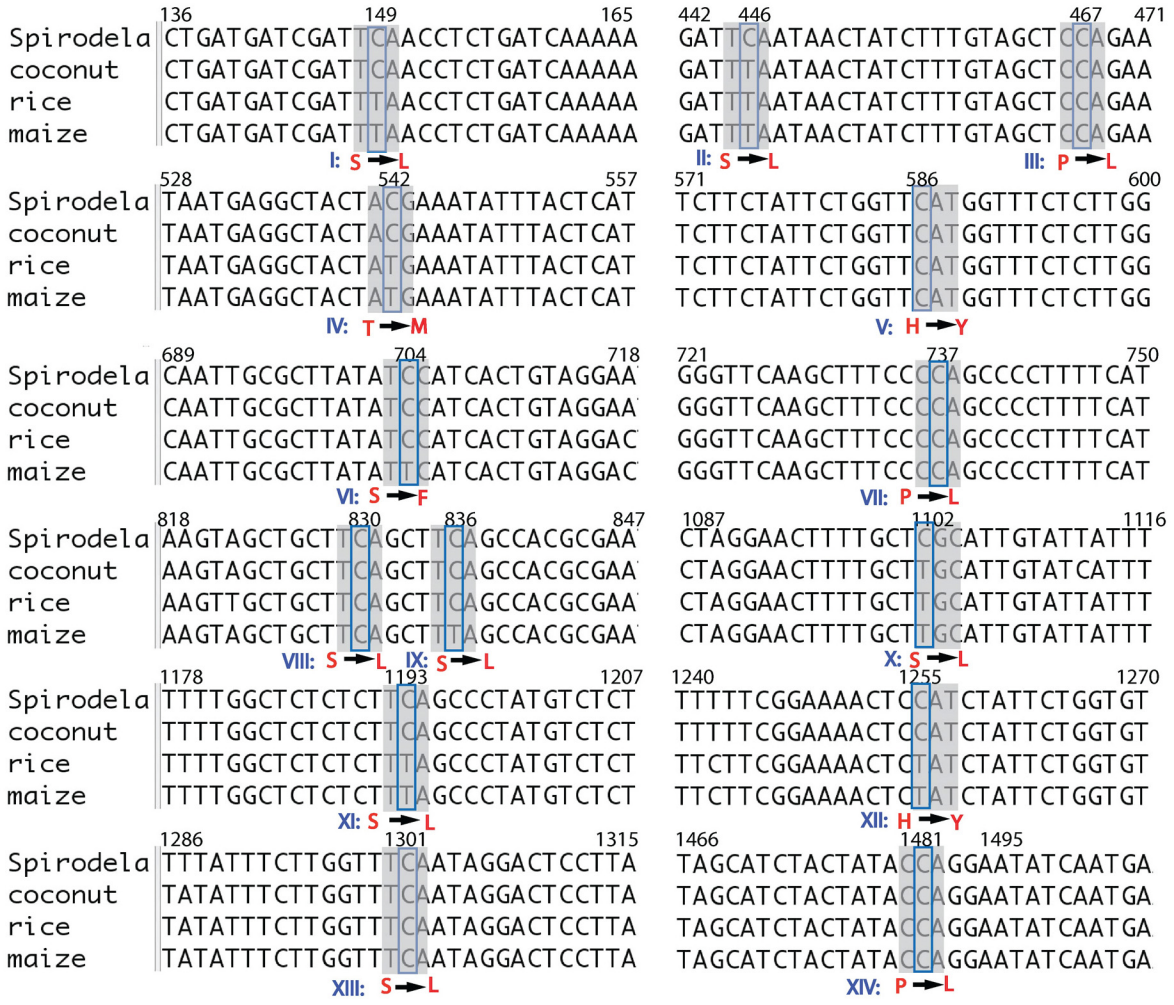
Alignment of editing sites in the *ndhB* gene. There were 14 edited sites identified in Spirodela *ndhB* coding region, plus another one from an intron which was not shown here. The amino acid substitutions caused by the editing events were marked with arrows. The locations in the CDS were listed on the top of alignment. All aligned sequences were obtained from the chloroplast genome sequence before RNA editing.

Noticeably, 31 out of 66 editing sites in Spirodela were from the *ndh* genes. A total of 15 out of 31 sites were from members of the *ndhB* genes (Table 1). Because of the well-studied and abundant editing sites within the plant kingdom [25,26], *ndhB* is a good example for the study of the conservation and evolution of RNA editing. We aligned and compared 14 editing sites except for the one in the intron of the *ndhB* coding region of Spirodela with coconut [16], rice [17] and maize [18] (Fig 2). All C-to-U transitions observed in ndhB transcripts occurred in either the first or second codon, thereby changing the amino acid identity. Six editing sites (III, V, VII, VIII, XIII and XIV) were conserved in Spirodela, coconut, rice, and maize. In contrast, two sites (VI and IX) were conserved in Spirodela, coconut and rice, but not in maize. However, four sites (I, IV, XI and XII) were only present in Spirodela and coconut. Contrary to the conserved sites, the newly identified sites II and X exhibited Spirodela-specific divergence. In all unedited locations, the T was already encoded at the DNA level, which eliminates the requirement for RNA editing.

**Fig 3:**
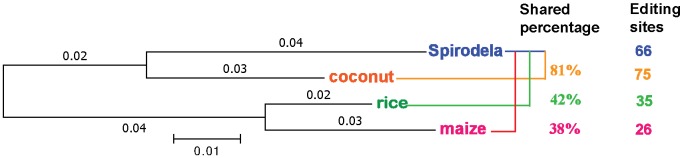
Phylogenetic tree of monocot representatives based on rbcL sequences. The tree was drawn by MEGA 6 maximum likelihood by using rbcL sequences of Spirodela, coconut, rice and maize. In the *ndh* gene family, Spirodela shares more RNA editing sites with coconut (81%) than rice (42%) and maize (38%).

In the phylogenetic tree drawn by *rbcL* alignment, Spirodela and coconut were sister groups, whereas rice and maize were sister species (Fig 3). Spirodela shared more editing sites with coconut than with rice and maize (Table 1). For example, in the well-studied *ndh* gene family of *ndhA*, *ndhB*, *ndhD* and *ndhF*, 21 (81%) out of 26 of the sites were common between Spirodela and coconut, whereas Spirodela shared only 11 (42%) of the sites with rice and 10 (38%) with maize (Fig 3). The observed distribution of shared editing sites was correlated with the phylogenetic tree: close sister species shared more common sites than distant ones. The conservation of RNA editing sites indicated that RNA editing originated from a common ancestor with many editing sites but followed by lineage-specific losses and gains during monocot evolution.

### RNA editing efficiency

**Fig 4:**
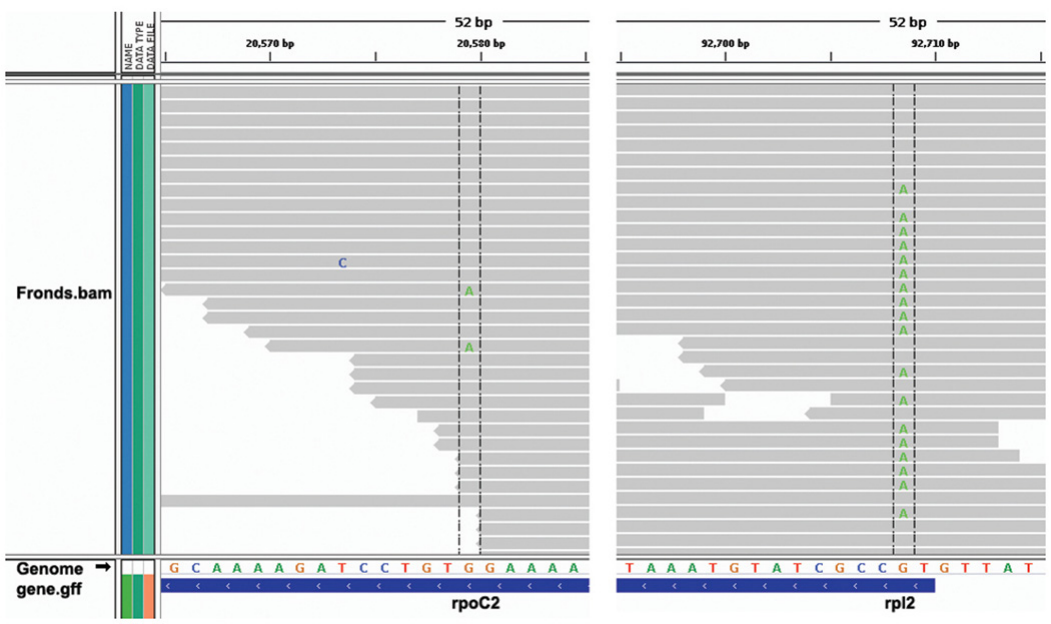
Visualization of RNA editing efficiency in the Integrative Genomics Viewer. Graphs were from the antisense strand of the reference genome. Here in gene *rpoC2*, two out of 33 reads were edited from C to U, whereas 31 reads were identical to the reference as C. In the *rpl2* gene, 18 out of 33 were changed into U. The examples indicate that RNA editing is partial. Not all the mapped reads were shown here due to high coverage.

RNA editing efficiency was counted by edited reads divided by total mapped reads (Table 1). We further validated editing efficiency by cloning the RT-PCR products and re-sequencing them with the CE platform. The consistent results suggested that RNA-seq analysis was sensitive and reliable for measuring the extent of RNA editing. We found that the individual RNA editing efficiency can vary dramatically from 6% to 100%, whereas the average value was 76%. As a consequence, RNA editing was incomplete to the degree of ~24% of total transcripts. For example, RNA editing efficiency was 7% for the *rpoC2* gene (position 20,579) and 44% for the *rpl2* gene (position 92,708) in fronds (Fig 1 and Table 1). Visualizing the mapping data in the Integrative Genomics Viewer explicitly showed that the RNA editing efficiency of rpoC2 was lower than rpl2 transcripts (Fig 4).

High gene expression and full editing efficiency would yield more edited transcripts and in turn more functional proteins. We found that chloroplast protein transcripts levels did not change significantly, when Spirodela development underwent turion formation. On the other hand, RNA editing efficiencies for five RNA editing sites (*ndhB*-15, *ndhD*-1, *rpoB*-1, *rps3*, *ndhC*-1) were significantly higher in fronds than in turions, whereas six sites (5'UTR *rps7*-1, *rps7*-2, *ndhC*-2, *ndhC*-3, *ycf3*-1, *ycf3*-5) were lower in fronds than in turions (Fig 1 and Table 2). The RNA editing efficiency of seven genes affected the yield of functional proteins because of 11 edited sites, but whether it plays a regulatory role during developmental change remains unclear.

**Table 2:** List of RNA editing sites with significant efficiency changes.

Gene	Genome Position	RNA edting efficiency in Frond	RNA edting efficiency in Turion
5'UTR rps7-1	105389	67%	90%
ndhB-15	102053	88%	62%
ndhC-1	56026	75%	65%
ndhC-2	55728	18%	30%
ndhC-3	55716	62%	92%
ndhD-1	130160	83%	61%
rpoB-1	28650	100%	81%
rps3	90143	54%	43%
rps7-2	104732	35%	65%
ycf3-1	49098	70%	94%
ycf3-5	47241	78%	92%

Seven genes with eleven RNA editing sites showed a significant change when growth is arrested at dormancy.

RNA editing could have significantly different editing efficiencies within the same transcript, for instance, for *ndhC* in a range of 30% ~ 92%, *ndhB* 8% ~ 100% and *ndhA* 25% ~ 95% (Fig 1 and Table 1), indicating that the individual RNA editing site is recognized by independent PPRs, a group of RNA editing factors. Furthermore, low-efficiency intergenic editing events (6% ~ 26%) and *rpoC2*-2 site (< 7%) appear not to be required for transcription or the function of the translated protein as coconut, rice, maize, Arabidopsis, tobacco and tomato develop normally even though they have the nucleotide “C” in this position (Table 1). The *rpoB* site is not edited in barley but edited in maize. Lack of RNA editing at this particular site does not seem to affect chloroplast function in barley [27]. The *rpoA* site is found edited in coconut and pre-edited in rice and maize. The *ndhB*-14 site is also pre-edited at the DNA level in tobacco and tomato. However, the conservation of RNA editing of *rpoA* and *ndhB*-14 in Spirodela could probably be due to the importance of their functions, in spite of the extremely low editing efficiency of < 11% and < 8%, respectively (Table 1).

## Discussion

### RNA-seq offers a method for qualifying and quantifying RNA editing

Transcripts from organellar genomes undergo extensive post-transcriptional processing, such as 5’- and 3’- end processing, RNA splicing and RNA editing [23]. RNA editing yields the conversion of cytosine (C) to uracil (U) nucleotides of mRNA transcripts. With prior knowledge of editing sites, primers are designed and RT-PCR products are processed to determine whether RNA editing occurs by comparing PCR products with the genomic DNA sequence. Such an approach, however, misses untranslated regions (UTRs), introns, and intergenic regions [24].

Furthermore, most editing sites are reported as fully edited, whereas partial editing is greatly underestimated. For editing efficiency of less than 10%, one has to sequence more than 10 clones to find one edited transcript when comparing cDNA with genomic sequences. Therefore, deep, strand-specific cDNA sequencing (RNA-seq) offers a new approach to identify all the potential RNA editing sites and quantify RNA editing efficiency, and to detect edited sites at very low efficiency [28].

Whereas the powerful technique of RNA-seq has been greatly utilized to study the nuclear transcriptome, it has not been widely applied to the organellar transcriptome because extracting transcripts from purified mitochondria and chloroplast is very time-consuming. Although one could sequence total RNA of both the nuclear and organellar genomes, the experimental method for preparing total RNA needs to be carefully considered. The reason is that organellar transcripts do not undergo polyadenylation like nuclear transcripts and do not have to be transported from one cellular compartment to another. Furthermore, post-transcriptional polyadenylation of organellar transcripts accelerates their degradation [29]. Therefore, the method of rRNA removal is preferred over the general approach using Oligo(dT)-based poly(A)+ enrichment for organelle transcript analysis. Compared to the isolation of RNA from purified organelles or the extraction of polyA mRNA for RNA-seq, rRNA removal by affinity is less biased, fast, and easily adapted to other plants.

### RNA editing evolution is of monophyletic origin in monocots

RNA editing is a system that exists in various land plant lineages, such as hornworts, ferns and seed plants but evolves very rapidly. Probably due to the limited verified editing sites, it was reported that the editing pattern was not correlated with the phylogeny of angiosperms [24][26], whereas other studies found that relatively closely related species shared more editing sites than distant species. For example, Nicotiana and Atropa from Solanaceae family shared 28 out of 31 RNA editing sites [30]. A total of 18 out of the 85 chloroplast-editing sites in seed plants were shared with either ferns or hornworts [7], indicating that the editing sites in seed plants could be remnants of the original editing system of land plants. After filling the phylogenetic gap with the editing sites data of a species of the order of the Alismatales, our results showed that 21 (81%) out of 26 sites of *ndhA*, *ndhB*, *ndhD* and *ndhF* transcripts were shared between Spirodela and its sister species coconut. In contrast, Spirodela shared only 11 (42%) of its sites with rice and 10 (38%) with maize, two more distantly related species (Fig 3 and Table 1), which is consistent with a monophyletic origin of RNA editing.

### Differential regulation of RNA editing in Spirodela

Partial RNA editing generates RNA polymorphism. In the *psbL* gene, RNA editing creates a translation initiation codon in tobacco. In another case, a chimeric gene conferring kanamycin resistance depended on ACG being edited into AUG. It was found that the unedited RNA could not be translated due to absence of an initiation codon [31]. However in yet another case, immunological analysis demonstrated that both unedited and edited *rps12* RNAs were translated in maize [32] and petunia mitochondria [33], resulting in the synthesis of polymorphic polypeptides. However, the translated proteins from unedited *rps12* transcript failed to assemble into ribosome in maize, whereas unedited *rps12* protein in petunia could integrate into ribosome, but whether it can function or not is not known.

In maize, the quantitative analysis for 10 plastid genes showed there were no expression differences in the green tissues including young leaf, old leaf, stems, and silks, except in roots and tissue-cultured cells [34]. Although developing turions enter a dormant state, their chloroplasts remain still quite active. They are functionally closer to amyloplasts, which are mainly responsible for the synthesis and storage of starch granules [12]. Like in green tissues of maize, we could not detect that chloroplast genes are differentially expressed between fronds and turions. However, seven genes with 11 RNA editing sites show a significant change of editing efficiency when fronds turn to turions. Interestingly, for these seven genes, it appears that RNA editing efficiency affects functional protein abundance more than the steady state level of mRNA. However, whether it plays a role in the morphological transition of Spirodela needs further investigations.

## Supporting Information

S1 Table Sequences mapped to the chloroplast genome. Samples contained four replicates of fronds and four replicates of turions [13]. Qualified total reads were based on the standard of minimum score of 20 and length of 70 bp. Reads were mapped back to the chloroplast genome. Mapped percentage was defined as mapped reads divided by qualified total reads.
(XLSB)

S2 Table Expression of chloroplast protein-coding genes. The significant change was considered when |Fold change| >2 and p-value < 0.05. The expression unit is FPKM.
(XLSB)

S3 Table Comparison of the number of RNA editing sites. RNA editing sites were compared in monocots including Spirodela, coconut, rice and maize. “NA” means the item was not studied.
(XLSB)

S4 Table RNA editing sites in non-coding regions. “Reference coverage” means the number of mapped reads identical to reference (four replicates are combined). “Edited coverage” means the number of mapped reads that have been edited (four replicates are combined). "Edit (%)” gives the percentage of RNA editing using the edited reads divided by total mapped reads.
(XLSB)
